# The recent landscape of cancer research worldwide: a bibliometric and network analysis

**DOI:** 10.18632/oncotarget.25730

**Published:** 2018-07-17

**Authors:** Bernardo Pereira Cabral, Maria da Graça Derengowski Fonseca, Fabio Batista Mota

**Affiliations:** ^1^ Instituto de Estudos em Saúde Coletiva, Universidade Federal do Rio de Janeiro, Rio de Janeiro, Brazil; ^2^ Instituto de Economia, Universidade Federal do Rio de Janeiro, Rio de Janeiro, Brazil; ^3^ Centro de Estudos Estratégicos da Fiocruz, Rio de Janeiro, Brazil

**Keywords:** cancer, oncology, scientific landscape, bibliometrics, social network analysis

## Abstract

The aim of this paper is to map the scientific landscape related to cancer research worldwide between 2012 and 2017. We use scientific publication data from Web of Science Core Collection and combine bibliometrics and social network analysis techniques to identify the most relevant journals, research areas, countries and research organizations in cancer scientific landscape. The results show: Oncotarget as the journal with most publications; a significant increase in China’s publications, reaching United States’ publications in 2017; MD Cancer Center, University of California and Harvard University as organizations with most publications; cell biology as the most frequent research area; breast, lung and colorectal cancer as the most frequent keywords; high density of co-authorship between organizations in the West, especially in the US, and low density between organizations in Asian and lower and medium income countries. Our findings can be used to guide a global knowledge platform guiding policy, planning and funding decisions as well as to establish new institutional collaborations.

## INTRODUCTION

Cancer cases worldwide reached 17.5 million cases in 2015, along with 8.7 million deaths. The incidence rate increased by 33% between 2005 and 2015 partly due to population growth and aging. Currently, cancer is the second cause of death worldwide and is expected to hit 27.1 million people by 2030 - especially in rich countries [1]. The main reasons are population aging and daily habits associated with the disease, such as smoking, alcoholism and sedentarism [1, 2]. The greatest impact of cancer will be in countries in the midst of economic and social transition [2].

Bibliometrics in studies related to cancer is relatively common in the literature. For example, bibliometrics has already been used to inform cancer research policy and spending [3] and path-breaking directions of nanotechnology-based chemotherapy and molecular cancer therapy [4]. It has also been used to analyze the current state or trends of research in specific types of cancer, such as lung cancer [5], esophageal and esophagogastric junction cancer [6] and triple negative breast cancer [7] . Specific topics within cancer research, such as the division between genders in scientific production, were also the subject of bibliometrics [8]. In this paper, we present the recent landscape of cancer research, as well as the relationships established in research (networks).

From this perspective, the aim of this paper is to map the scientific landscape related to cancer research worldwide between 2012 and 2017, combining bibliometrics and Social Network Analysis (SNA) techniques. To map the scientific landscape related to cancer, we used data from scientific publications available in the Web of Science Core Collection (WoS), Thomson Reuters. The paper describes the comprehensive research status of the cancer field by analyzing the quantity of publications, main journals, frequent research areas and most scientifically productive countries and organizations. It also explores the global cooperation network of these countries and organizations, identifying most central players, and the association of different research topics, highlighting the one that is mostly associated with innovative efforts in the field. It aims to generate evidence that could ultimately inform managers, researchers and policy makers, supporting decision-making, R&D planning and financing strategies.

## RESULTS AND DISCUSSION

**Figure 1:**
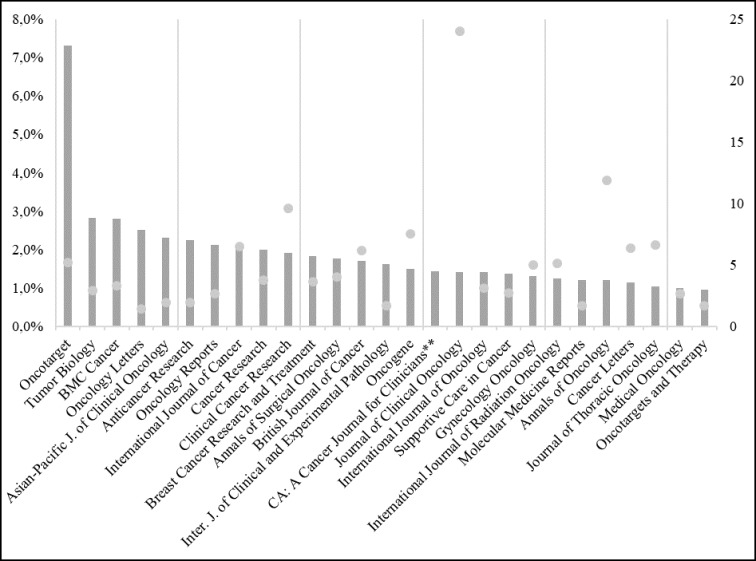
Most frequent journals and its impact factors

Figure 1 presents journals with at least 1% of publications between 2012 and September 2017 and its Impact Factors for the 2016/2017 period. The number of articles published in these journals highlights those where the cancer-related scientific community has preference in publishing. Oncotarget led with 7575 articles related to cancer (7.3% of the total), followed by Tumor Biology and BMC Cancer (2.8% each).

Much of the journals are focused entirely on cancer. Molecular Medicine Reports, whose scope includes different topics in molecular medicine (pharmacology, pathology, genetics, neuroscience, infectious diseases, molecular cardiology, and surgery molecular) was one exception [9]. CA: The Cancer Journal for Clinicians’ had the biggest Impact Factor (187,040).

**Figure 2:**
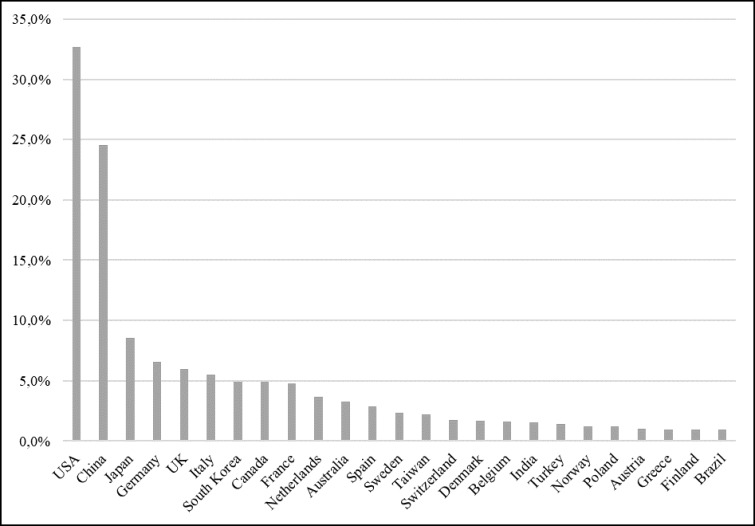
Countries with most publications

Figure 2 shows the ranking of countries with at least 1% of overall publications. Data refers to authors’ institutional affiliation and, since papers are usually co-authored, the number of records is greater than the total of papers. The United States (USA) stands out due to the volume of its production (32.7% of all publications) followed by China (24.5% of total publications). Together, these two countries account for more than half of all scientific publications (8.6% of all publications). Countries with high income and human development index are the more productive, with the exception of China, India, Turkey and Brazil.

**Figure 3:**
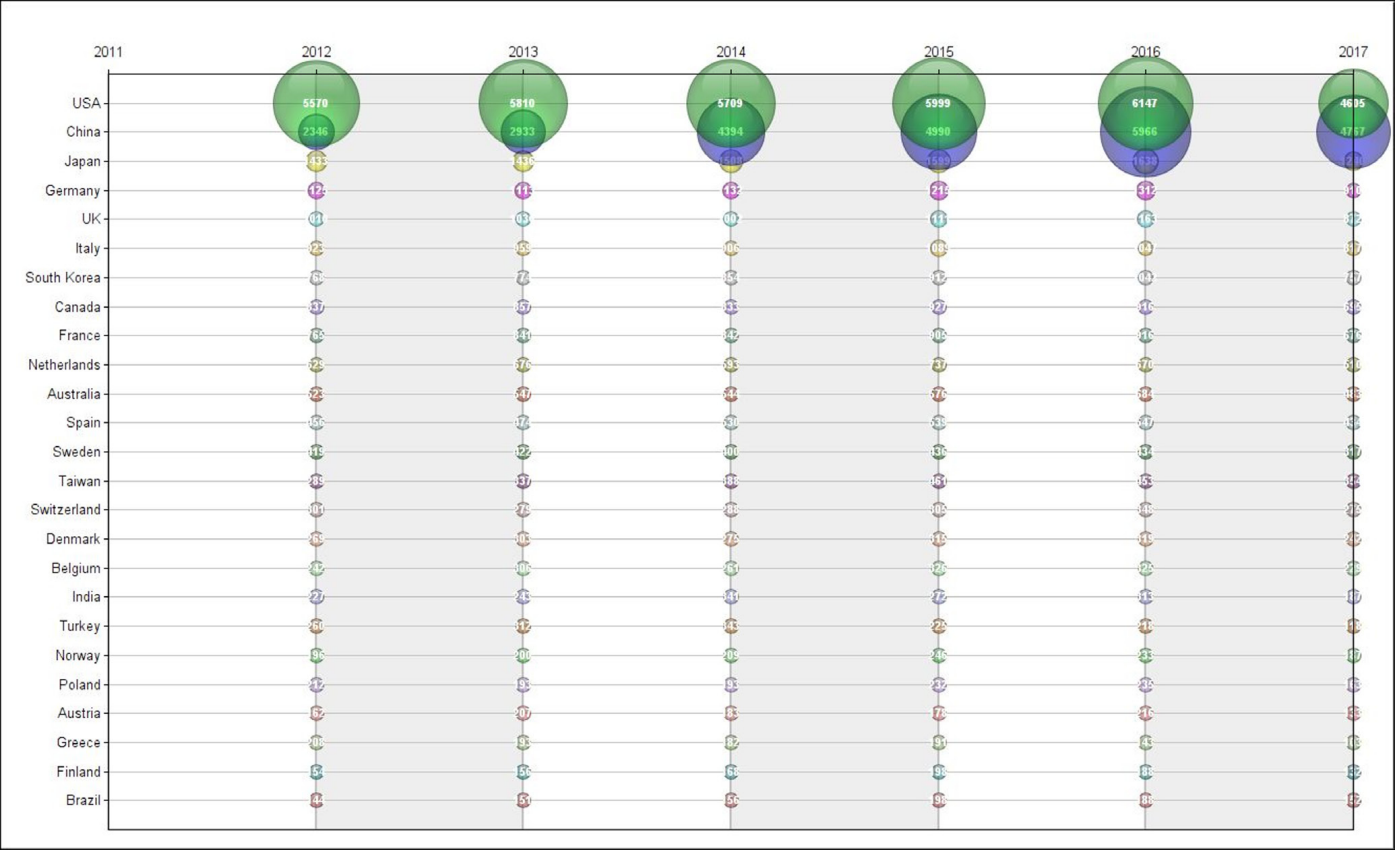
Number of cancer publications by country

Figure 3 shows the evolution of publications among countries with at least 1% of overall publications. United States’ lead in publications over the past five years is noteworthy. In the other hand, China significantly increase in its number of publications is also remarkable - jumping from 2346 in 2012 to 5966 in 2016 (a 154% expansion). In 2017, China’s publication surpassed the total of American publications in the same year (4767 and 4605, respectively). All other countries at the top of the publications ranking had an increase in the number of publications. China’s publications over time should be analyzed taking into account important aspects about this country. Cancer has been the leading cause of death in this country since 2010 [10] and the country’s efforts to deal with this public health problem has been highlighted in the country’s latest five-year plan - which includes a substantial increase in investments in research [11].

**Figure 4:**
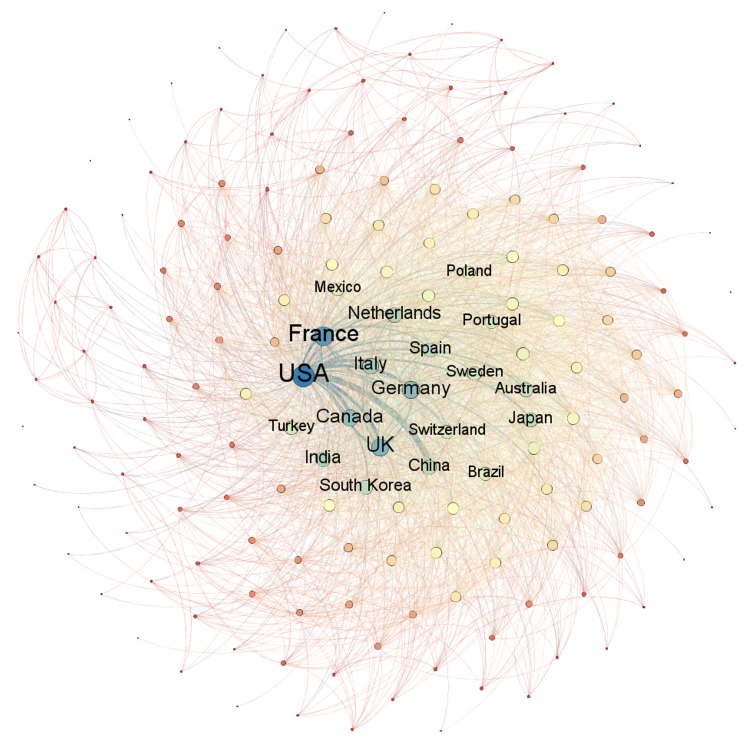
Network of countries with publications on cancer

Figure 4 complements the previous one presenting the network of countries with publications on cancer. The network contains all countries with publications of authors linked to an organization of that country. The country’s name is highlighted for those who had more partnerships - in this case, co-authored publications. We built the network based on the degree of entry of each country into the co-occurrence matrix of publications. In other words, the countries are organized in the network according to the total of partnerships. The size of each node highlights the countries with more partnerships and the thickness of each edge highlights the amount of partnerships between two countries.

The network highlights the prominent role of the United States. This country is the one with the biggest node, which means it is the one with more partnerships. Japan and China are respectively second and third in the ranking, but the size of its nodes show a lower degree of partnership than other countries with fewer publications. For example, France and the United Kingdom are countries with significantly fewer publications than these two, but with higher degree of entry into the network. Data suggests that for China and Japan there are more partnerships between organizations within the country than with organizations in other countries.

**Figure 5:**
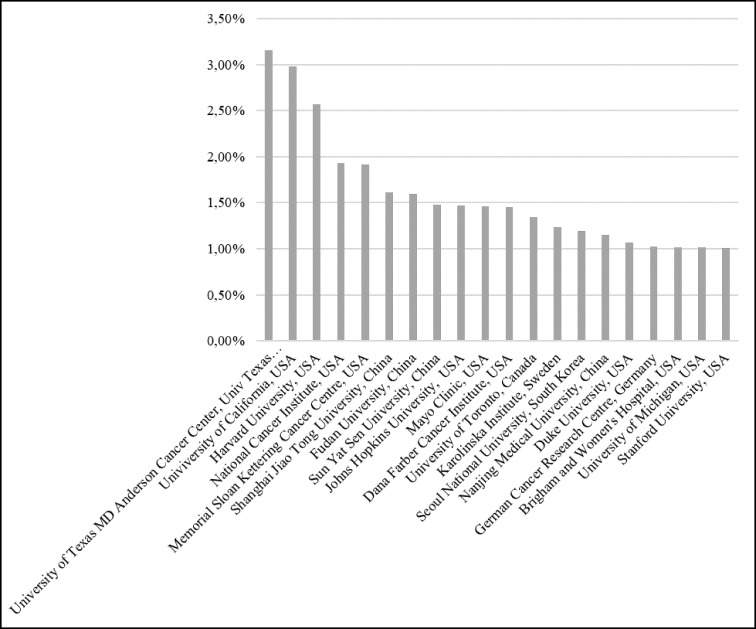
Organizations with most publications

Figure 5 presents the organizations with participation of at least 1% in the total of publications. There are twenty organizations from six different countries: (1) the United States, with twelve organizations, (2) China, with four organizations and (3) Canada, Sweden, South Korea and Germany, with one organization each. Although it resembles the countries’ ranking, one can see some important distinctions. China is close to the United States in terms of countries’ publications, but the relevance of the United States stands out when we individualize the analysis by organization. The sum of the first three organizations in the ranking, all American, represents approximately 10% of the total scientific publications on cancer in the period.

Another highlight among organizations is the Karolinska Institutet, in Sweden. This organization is the thirteenth in this ranking, with far superior representation compared with Sweden amongst the total publications of the countries. The Karolinska Institutet is responsible for more than half of the cancer publications in Sweden and publishes slightly less than the main China’s cancer organizations. Founded in 1810, this organization is one of the world’s leading medical organizations with 80 percent of its revenue exclusively dedicated to research [12].

**Figure 6:**
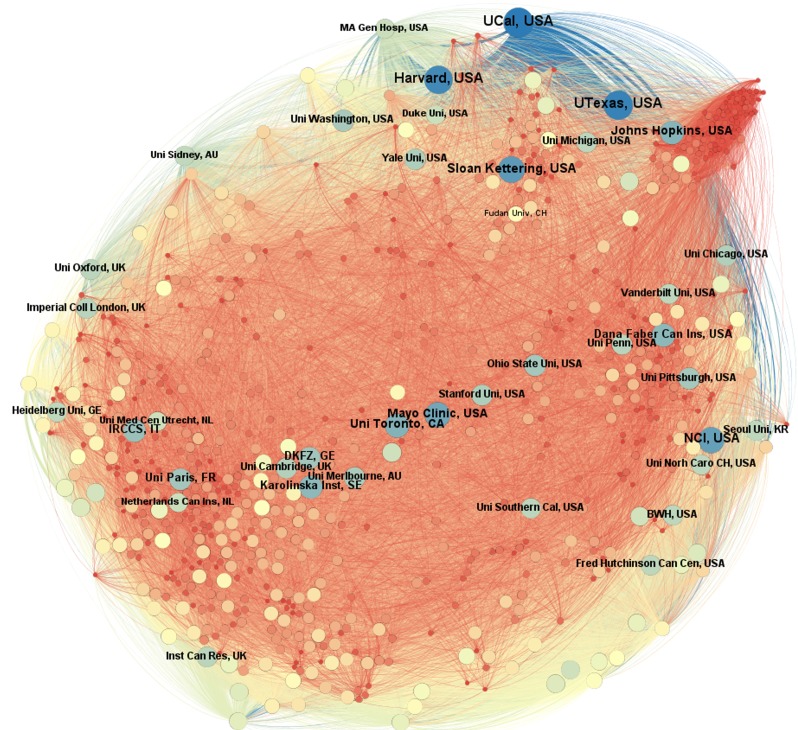
Network of organizations with publications on cancer

Figure 6 evidences how the organizations interacted. Like the previous one, we organized this network according to the degree of entry of each node. More precisely, the number of articles in authorship with authors from other organizations. Those organizations with more partnerships were highlighted by adding its name in the figure. Fudan University, the Chinese organization with more partnerships, was also highlighted, even with far less degree of entry compared to the other named organizations. The main institution in terms of partnerships were the ones located in countries with the largest number of publications on cancer. The network algorithm used (Fruchterman Reingold) allows us to visualize some groupings of organizations that interact more among each other, as in the case of grouping at the top of the image, which counts only with American organizations. There is also a grouping of European organizations on the lower left side of the figure and another grouping of American organizations on the right side, which includes the nation’s leading cancer organization, the National Cancer Institute (NCI). To facilitate visualization, the MD Cancer Center of the University of Texas was grouped with the other organizations that are also part of the so-called Texas System. These organizations are grouped by the name ‘UTexas’.

Again, the comparison of total publications and partnerships in China has different results. Although this country has four of the twenty organizations with more publications on cancer, the number of partnerships is significantly lower than some organizations with much less publications. One notes that the smaller scale of partnerships in Chinese organizations is not only for partnerships between different countries, but also within the country itself. The data suggest that the high volume of publications in this country is more often the result of intrainstitutional efforts.

**Figure 7:**
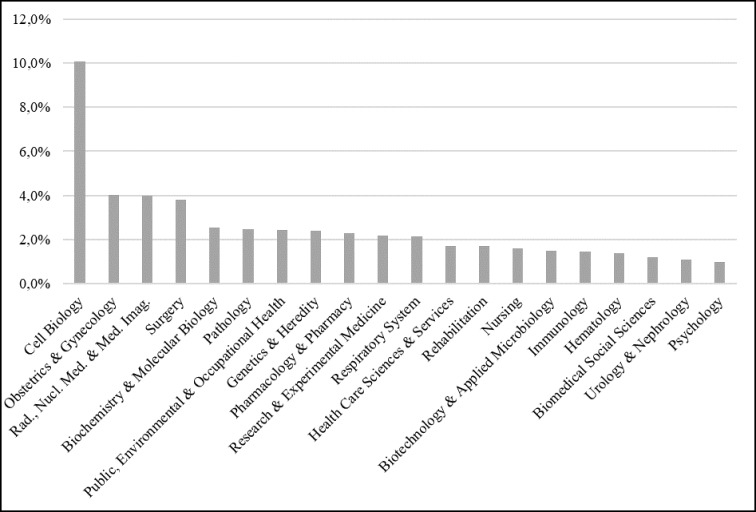
Research areas with most publications on cancer

Another relevant category to map scientific publications on cancer is the research area. Thomson Reuters’ team reads indexed articles in the Web of Science database and assigns one or more research areas to each of them. The database is currently divided into 151 research areas, which come from five major research areas: (1) Biomedicine and Life Sciences, (2) Physical Sciences, (3) Technology, (4) Arts and Humanities, and (5) Social Sciences [13]. Figure 7 shows the research areas with at least 1% of total publications.

The most prominent research areas can be divided into large areas of knowledge segmented by cancer sites (obstetrics and gynecology, respiratory system, etc.), types of treatment (surgery, radiotherapy, immunology, etc.) and types of scientific knowledge (cell, molecular biology, etc.). One can notice the prominence of research related ​​to cellular biology in relation to the others, with more than 10 thousand publications indexed. The other research areas represents the main modalities of cancer diagnosis and treatment, except immunology, which is only in the sixteenth position. Although this modality of treatment is currently considered one of the most modern on cancer care, traditional approaches to treatment are still those that concentrate the largest amount of publications.

**Figure 8:**
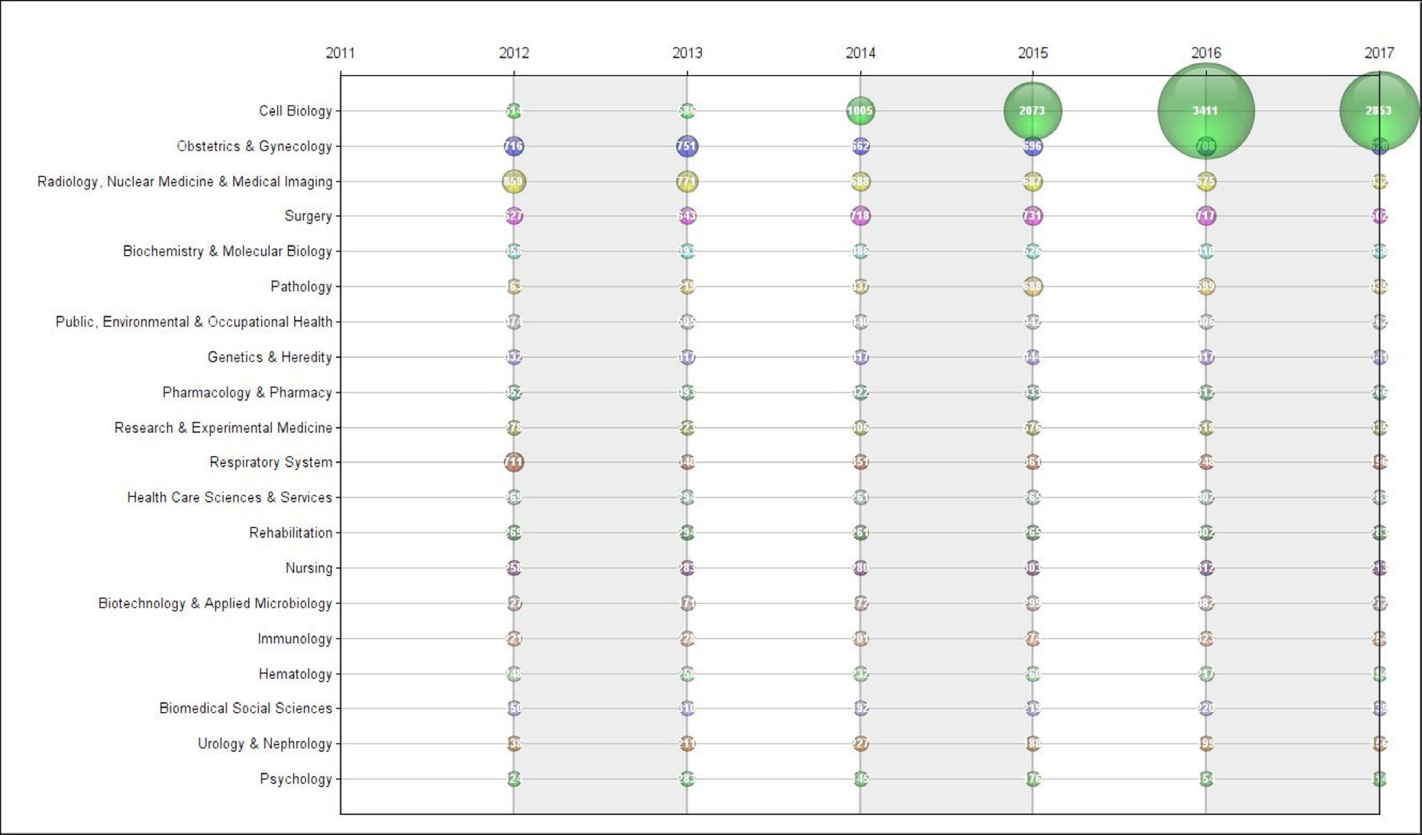
Publications on cancer by research area

Figure 8 breaks down research area publications over time. One can see the rise in the number of publications in the area of ​​cell biology. It jumps from 514 publications in 2012 to 3411 publications in 2016 (or 564%). Other areas also grew in this period, such as surgery, pathology and research and experimental medicine. At the same time, the areas of ‘radiology, nuclear medicine and medical imaging’ and the respiratory system saw a reduction in the number of publications.

Research and experimental medicine is also highlighted. According to the Thomson Reuter classification, this category includes papers that analyze or promote the creation of techniques considered extremely innovative. Most of the work involves research at an early stage of development, that is, still distant from the market and with great uncertainty associated with their future. This category increased steadily in this period (86% increase). The search for innovative interventions on cancer care has been a trend in the industry and academia for some decades and the funding of these researches has been growing substantially [14]. Research and development in oncology is known in the industry as having one of the lowest success rates (and higher costs) among the most prevalent diseases in the global epidemiological profile. Usually, for each new molecule entering clinical study the likelihood of becoming a commercially viable product is only 5% [14]. Still, by 2016 there were 544 companies in the world with late-stage clinical research and at least 631 new molecules at the developmental stage [15].

**Figure 9:**
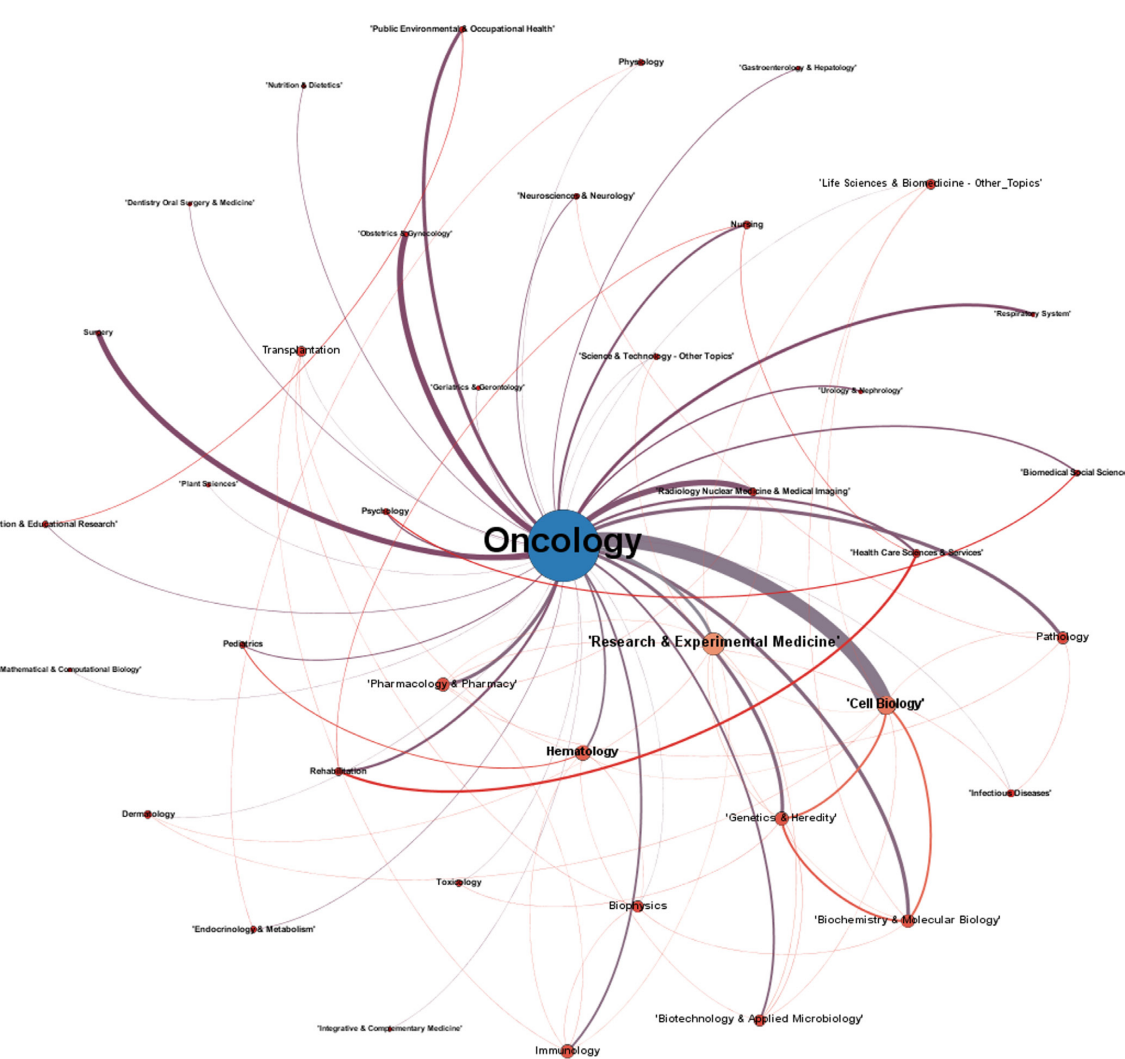
Research areas network for publications

Figure 9 complements the previous analysis with the network of research areas. The size and relevance of oncology is an obvious consequence of the search strategy. All nodes were named and the font size is related to the frequency of co-occurrence of each area with the others. The network of research areas shows the relevance of cellular biology since the edge between the two areas is thick. The area of research and experimental medicine’ has more co-occurrence with the others despite the smaller volume of publications.

**Figure 10:**
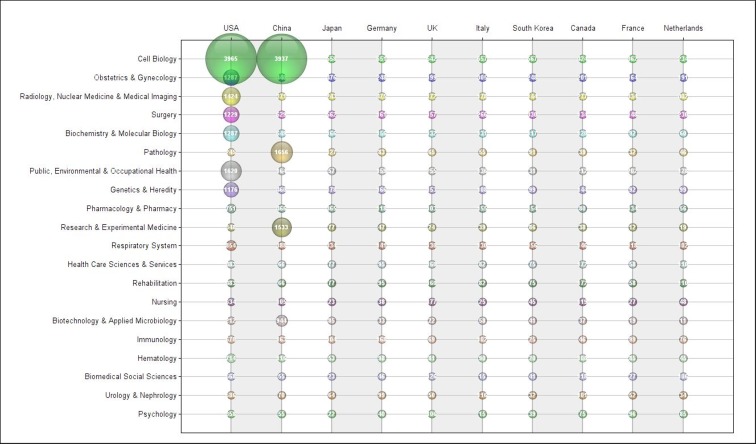
Number of publications in each country according to the research area

Figure 10 provides additional information by showing research area publications by country over time. It mainly highlights the distribution of the United States and China in terms of publications. In addition to a large number of publications in the area of ​​molecular biology, one can note that there is a specialization in these countries for the other eight areas of research with more frequency of publications. The United States focuses its research on: (1) obstetrics and gynecology, (2) radiology, nuclear medicine and medical imaging, (3) surgery, (4) biochemistry and molecular biology, (5) public, environmental and occupational health and (6) genetics and heredity. On the other hand, China concentrates publications in: (7) pathology and (8) research and experimental medicine. It was not possible to look deeply into China’s indexed publications in research and experimental medicine, but it looks like this country is the most dedicated to radical scientific advances in the last five years. In fact, the Chinese government funding strategy points to a hunt for innovative interventions on cancer care [16].

**Figure 11:**
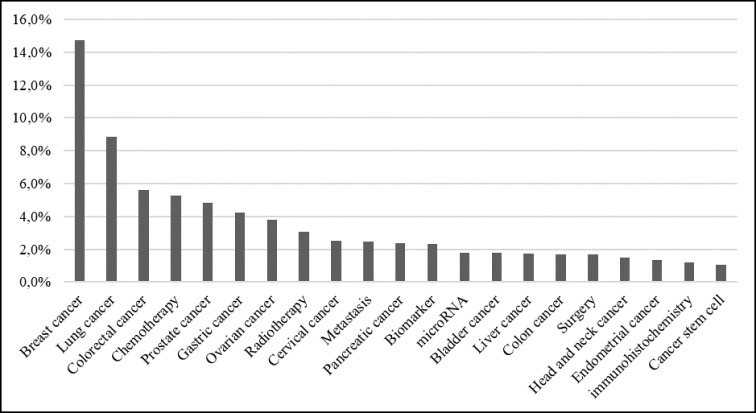
Most frequent keywords

Figure 11 presents the ranking of the main keywords, apart from the term ‘cancer’, which was the most frequent for obvious reasons. This ranking contains all terms with at least 1% of share in total keywords. We can divide the terms between specific types of cancer, therapies and technologies. The three most frequent keywords are breast cancer, lung cancer and colorectal cancer. Besides these three specific types of cancer are also among the most cited: prostate, gastric, cervical, pancreatic, bladder, liver, colon, head and neck and endometrial cancer in this order. Chemotherapy, radiotherapy and surgery are also prominent - those are the most common conventional treatments for cancer. The terms metastasis, biomarkers, microRNA, immunohistochemistry and stem cell carcinogen complete the ranking of the most frequent keywords in the documents analyzed.

The prominence of breast cancer among the keywords probably relates to its relevance in the global epidemiological profile. Breast cancer accounts for 6% of cancer deaths and 12% of new cases recorded in 2012. Although there is more up-to-date data for specific countries, the figures for the total number of cancer cases in the world, which is compiled by the Agency International Cancer Research (IARC) of the World Health Organization, are only updated to 2012. Although male breast cancer exists in a smaller proportion, if only cancers in women are considered, this is the one with the highest mortality rate (14.7% of total cancer deaths) and a higher incidence (25.2% of the total new cases) in 2012. Breast cancer is the most common cancer in women in 140 countries and the most frequent cause of cancer mortality in 101 countries [17].

Besides breast cancer, lung and colorectal cancers also have prominence in the global epidemiological profile. Lung cancer is the cancer with the highest rates of mortality and incidence among all cancers in the year 2012, while colorectal cancer is the third in terms of incidence and the fourth in terms of mortality. An analysis of incidence and mortality numbers on cancer shows that the publications are much more consistent with disease incidence rates than with their mortality rates.

The total amount invested in research in each of these areas may also be another factor to explain this distribution. A proxy for these investments can be found in the total investments made by the National Institutes of Health (NIH), the leading research funding organization in the United States. Breast cancer is the one with the highest investment among all other cancers, with almost double the funding compared to second-placed pediatric cancer ($ 656 million and $ 351 million in 2016). Lung and colorectal cancers are also cancers with a lot of funding in recent years [18].

## MATERIALS AND METHODS

To map the scientific landscape related to cancer, we used data from scientific publications available in the Web of Science Core Collection (WoS), Thomson Reuters, and combined bibliometrics and social networks analysis techniques. In WoS, the following search strategy was used:

((ti=(cancer* or neoplasia* or neoplasm* or tumor*) and su=(oncology))) AND DOCUMENT TYPES: (Article) Indexes=SCI-EXPANDED Timespan=2012-2017

It was decided to restrict the search only to articles because these documents meet higher quality standards than other types of scientific dissemination materials [19]. In order to collect publications related to the natural sciences - especially biomedical publications - the search was restricted to the SCI-EXPANDED index. The search was conducted on October 9, 2017 and obtained 105,512 records, which were imported (into a text file) to the proprietary software VantagePoint 10.0 for treatment and analysis.

Duplicates were removed using the ‘ISI Unique Article Identifier’. After that, the number of documents was reduced to 89,067. The fields ‘author affiliations (Organization and City and Country)’ were normalized using the general fuzzy logic from Vantage Point’s list cleanup tool as well as manual cleaning. The keywords were grouped according to the type of cancer, form of treatment or technology. The rankings were produced in VantagePoint and exported to Microsoft Excel for building the graphics. Bubble charts were the only ones produced in VantagePoint itself.

The networks were produced using the software Gephi 0.9.1 from co-occurrence matrices generated in Vantage Point. We used the Fruchterman Reingold (FR) algorithm, which assumes the existence of groups or clusters within the network [20]. For each of these networks, their degree of centrality was used as reference. The degree of centrality is based on the number of nodes directly connected to other nodes in the network and is adequate to represent the influence of each node in the network [21].

## CONCLUSIONS

Our paper provides interesting insights to cancer scientific landscape. It provides a concise view of the scientific knowledge distribution through thousands of publications around the world and the key elements for understanding the dynamics of this knowledge. Although the timespan was only five years, it was enough to show trends among research areas, and specially China’s rise as an important contributor in the generation of knowledge. Also, the networks showed how cancer is the subject of interinstitutional efforts worldwide - with a higher density among Western countries.

## SUPPLEMENTARY MATERIALS










