# Supplementary material for: The recent landscape of cancer research worldwide: a bibliometric and network analysis
Source: Oncotarget. 2018 Jul 17;9(55):30474–84. doi: 10.18632/oncotarget.25730 (PMC6078146; doi:10.18632/oncotarget.25730)
Supplement: Supplementary file 1 [file oncotarget-09-30474-s001.pdf]

The recent landscape of cancer research worldwide: a bibliometric and network analysis

SUPPLEMENTARY MATERIALS

For Supplementary Dataset 1 see attached excel file in Supplementary Files.

For Supplementary Dataset 2 see attached excel file in Supplementary Files.

For Supplementary Dataset 3 see attached excel file in Supplementary Files.

For Supplementary Dataset 4 see attached excel file in Supplementary Files.
